# Supplementary material for: Pilot plant study on nitrogen and phosphorus removal in marine wastewater by marine sediment with sequencing batch reactor
Source: PLoS One. 2020 May 19;15(5):e0233042. doi: 10.1371/journal.pone.0233042 (PMC7236998; doi:10.1371/journal.pone.0233042)

S1. Fig. The locations of marine-sediment sampling site (a) and pilot plant setup (b) in the South Korea (c).

(a)


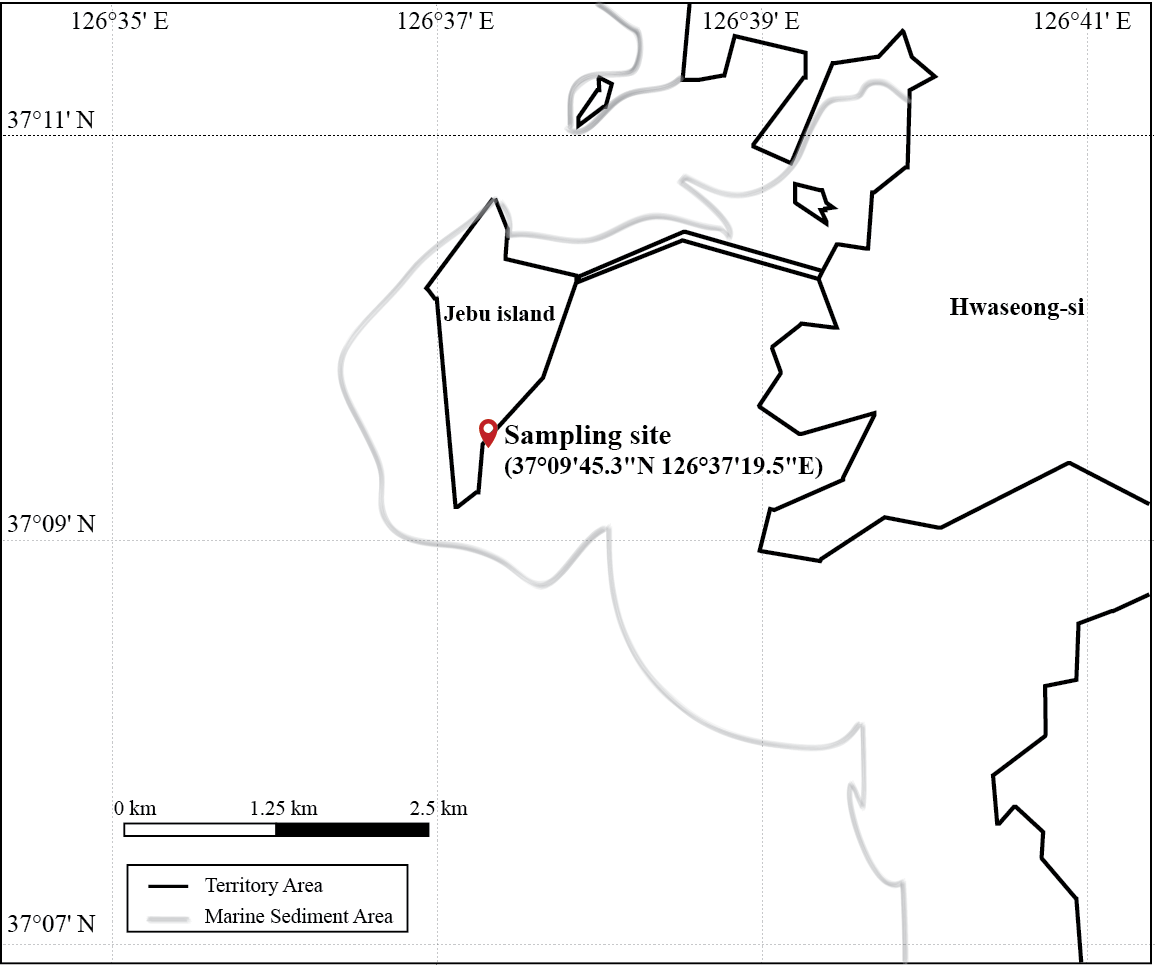


(b)


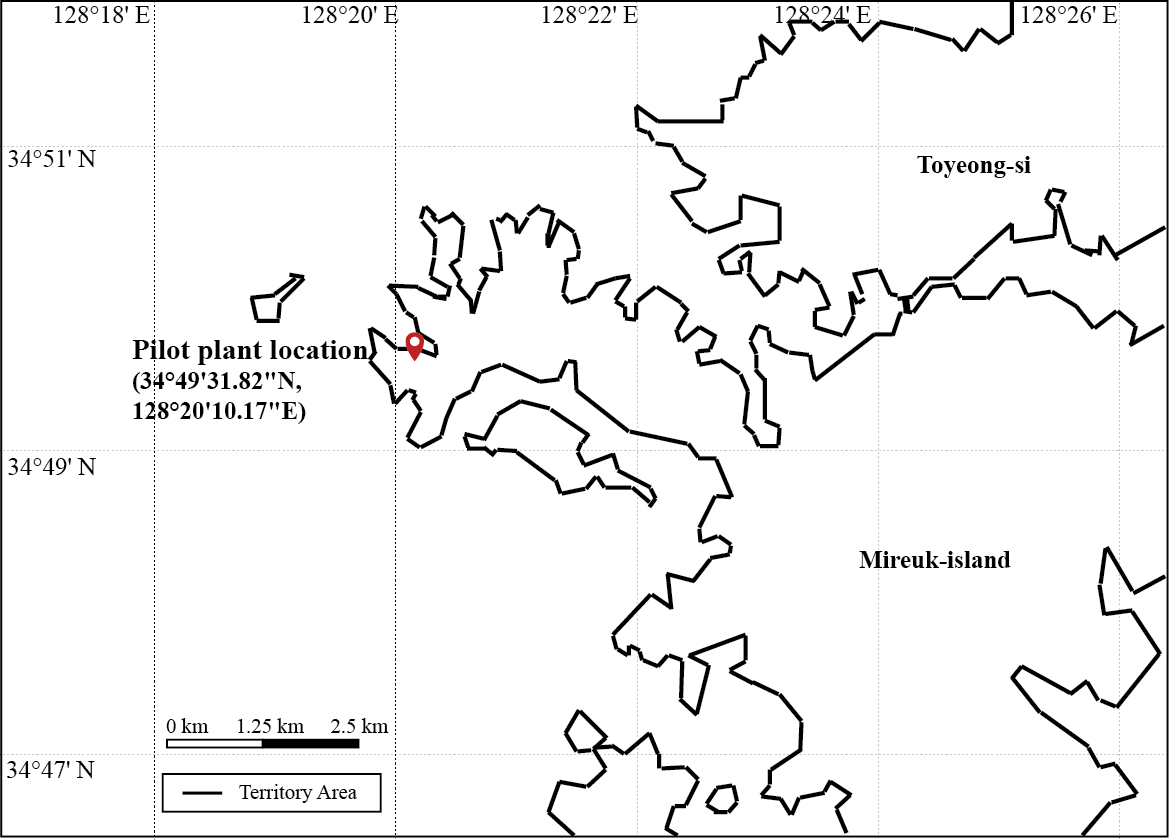


(c)


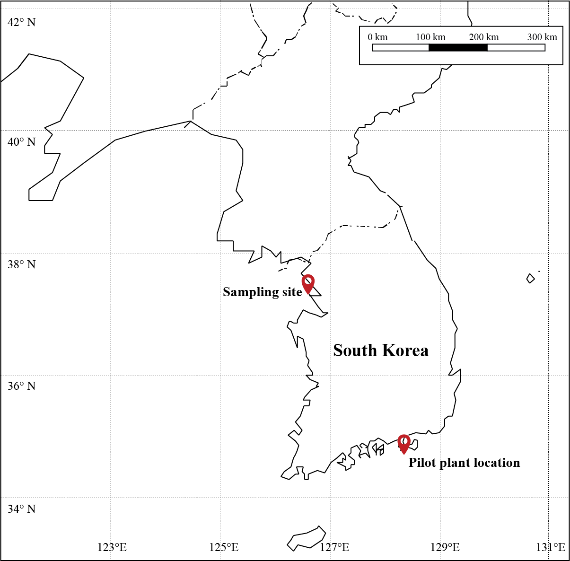

Supplement: S1 Fig — The locations of marine-sediment sampling site (a) and pilot plant setup (b) in the South Korea (c). (DOCX) [file pone.0233042.s001.docx]
