# Supplementary material for: Pilot plant study on nitrogen and phosphorus removal in marine wastewater by marine sediment with sequencing batch reactor
Source: PLoS One. 2020 May 19;15(5):e0233042. doi: 10.1371/journal.pone.0233042 (PMC7236998; doi:10.1371/journal.pone.0233042)
Supplement: S2 Fig — (DOCX) [file pone.0233042.s002.docx]

S2. Fig. Analytical data of environment factors such as temperature, pH, and salinity in marine wastewater for the pilot plant-scale SBR treatment system during all operation period.
