# Supplementary material for: Pilot plant study on nitrogen and phosphorus removal in marine wastewater by marine sediment with sequencing batch reactor
Source: PLoS One. 2020 May 19;15(5):e0233042. doi: 10.1371/journal.pone.0233042 (PMC7236998; doi:10.1371/journal.pone.0233042)
Supplement: S3 Fig — Treatment of organic carbon (CODCr, (a)), total nitrogen (b), total phosphorus (c) by the eco-friendly high efficiency marine sludge (eco-HEMS) and the aerobic granule sludge (AGS) in the pilot plant-scale SBR biological treatment system during operation period. (DOCX) [file pone.0233042.s003.docx]

S3. Fig. Treatment of organic carbon (COD_Cr_, (a)), total nitrogen (b), total phosphorus (c) by the eco-friendly high efficiency marine sludge (eco-HEMS) and the aerobic granule sludge (AGS) in the pilot plant-scale SBR biological treatment system during operation period.

(a)

(b)

(c)
