# Supplementary material for: Pilot plant study on nitrogen and phosphorus removal in marine wastewater by marine sediment with sequencing batch reactor
Source: PLoS One. 2020 May 19;15(5):e0233042. doi: 10.1371/journal.pone.0233042 (PMC7236998; doi:10.1371/journal.pone.0233042)

S5. Fig. The analytical profile of eco-HEMS applying SBR treatment system in winter when COD: N: P ratio was 300:5:1. (a), environment factors; (b), COD_Cr_ and MLSS, (c), NH_3_-N, NO_3_^-^ -N, and T-N; (d) PO_4_^3-^ -P and T-P. SBR stages consisted of follow 4 stages: I, influence; II, aeration and mixing reaction; III, anaerobic and settlement, IV, decant-effluence and idle.

(a) (b)


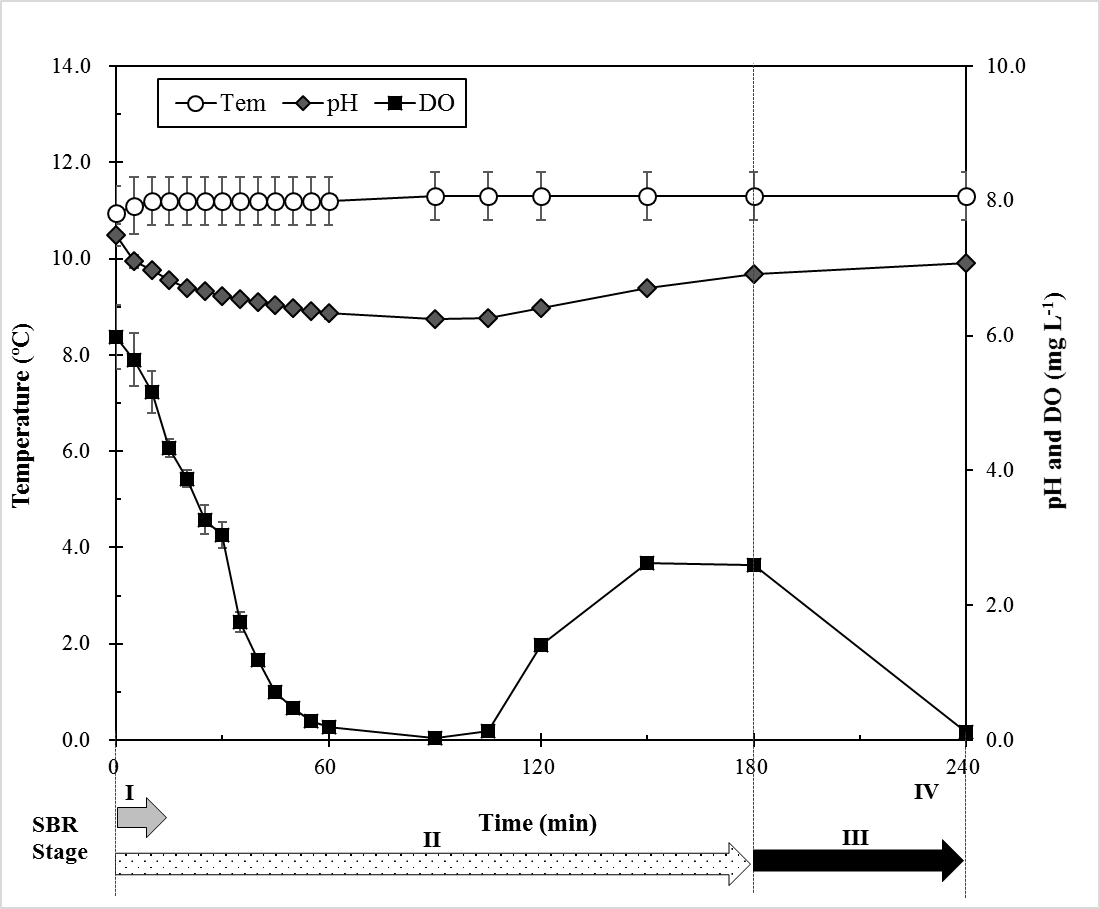

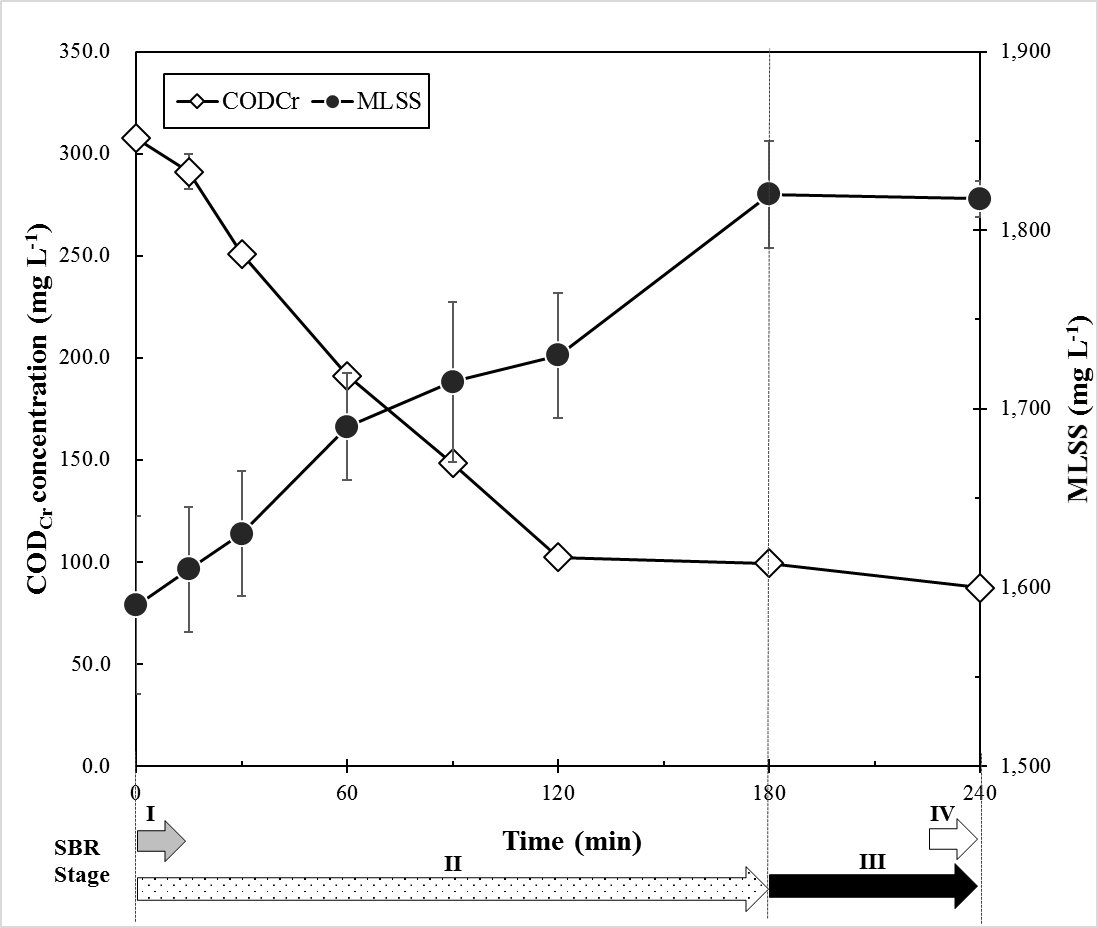


(c) (d)


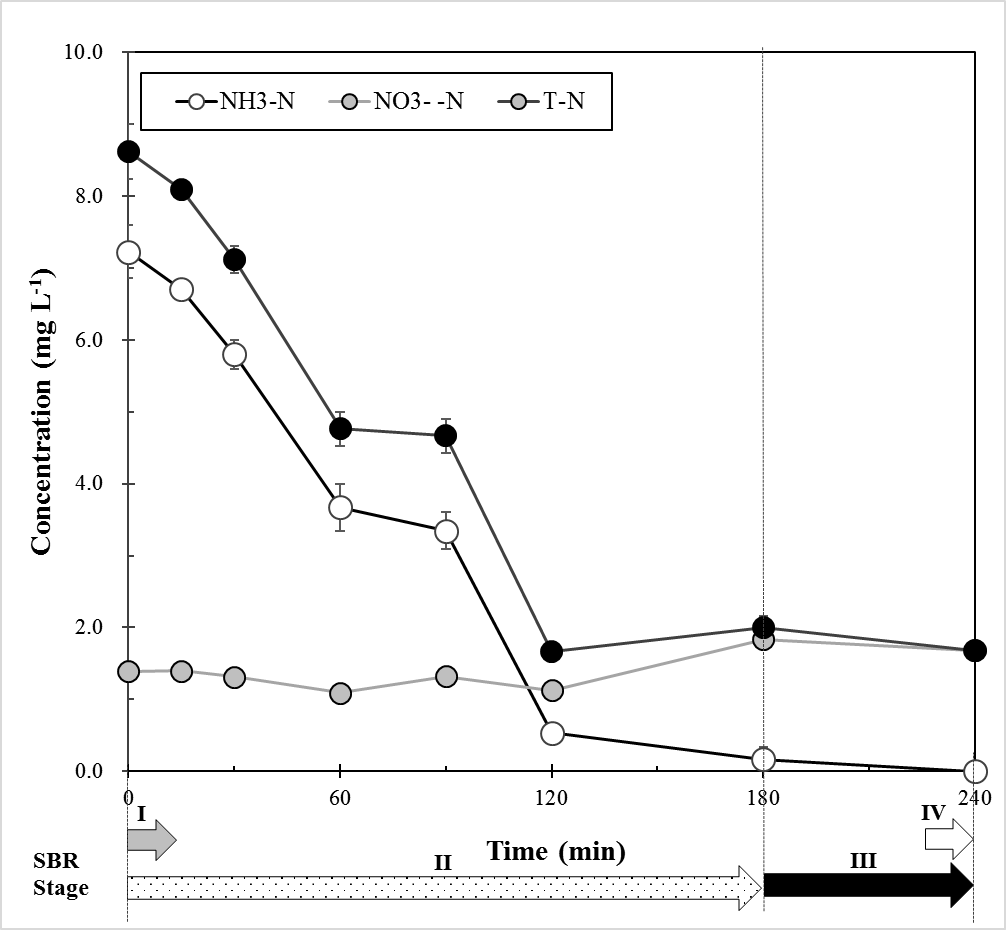

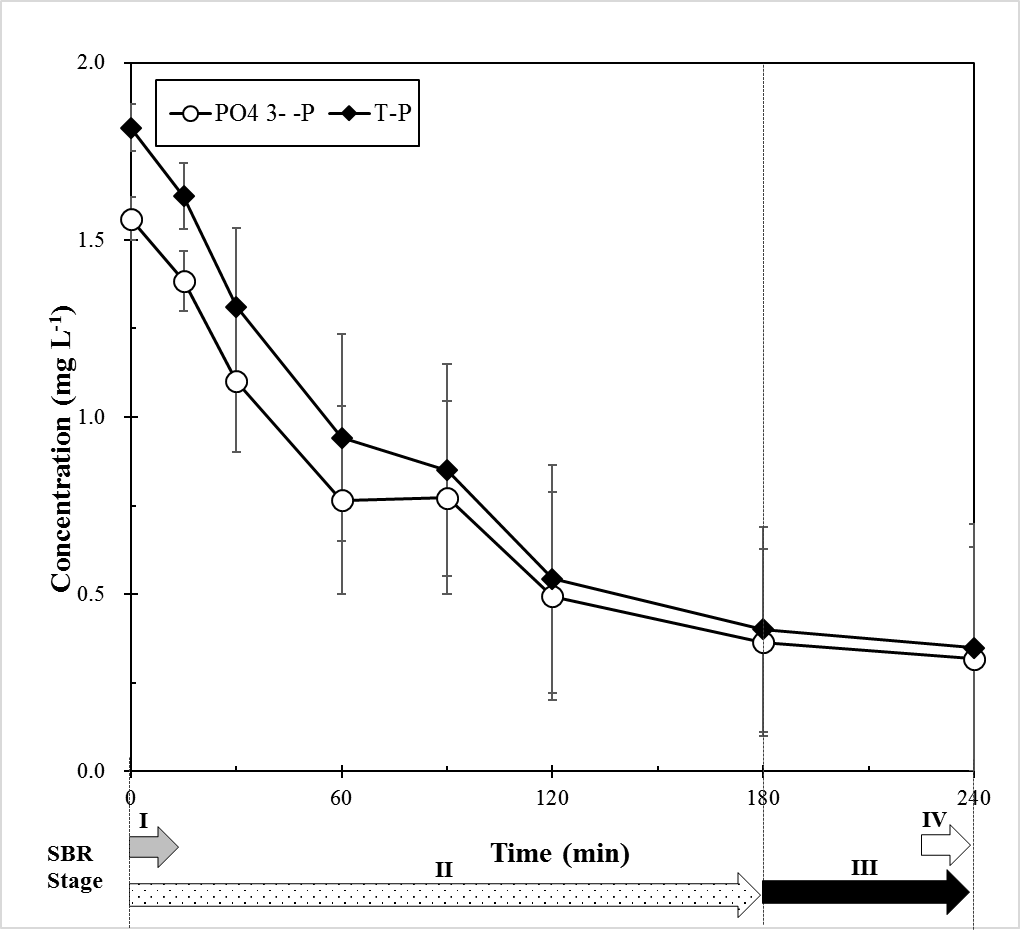

Supplement: S5 Fig — (a), environment factors; (b), CODCr and MLSS, (c), NH3-N, NO3- -N, and T-N; (d) PO43- -P and T-P. SBR stages consisted of follow 4 stages: I, influence; II, aeration and mixing reaction; III, anaerobic and settlement, IV, decant-effluence and idle. (DOCX) [file pone.0233042.s005.docx]
