# Supplementary material for: Pilot plant study on nitrogen and phosphorus removal in marine wastewater by marine sediment with sequencing batch reactor
Source: PLoS One. 2020 May 19;15(5):e0233042. doi: 10.1371/journal.pone.0233042 (PMC7236998; doi:10.1371/journal.pone.0233042)

S8. Fig. The principle component analysis (PCA) with PC plots for bacterial communities during adaptation period from the marine sludge to eco-HEMS.

(a) PCA analysis based on PC1 vs PC2, (b) PCA analysis based on PC1 vs PC3, (c) PCA analysis based on PC3 vs PC2; 0 week (⚫), 1 week (◼), 2 week(▶), 3week (▲),4week (◀), 5 week (▼).

(a) (b) (c)


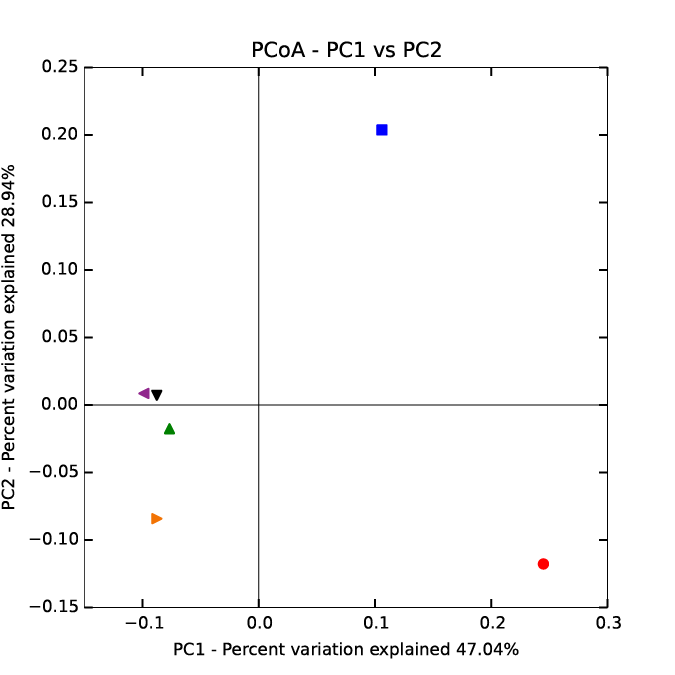

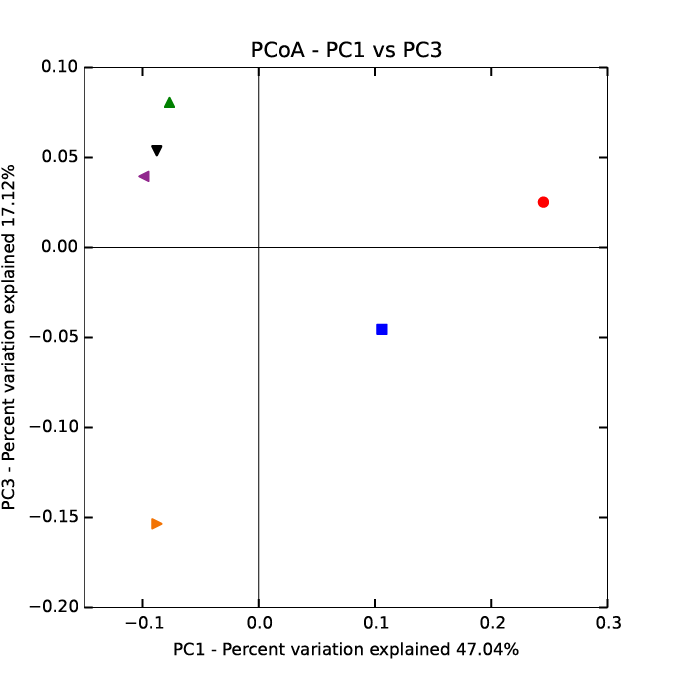

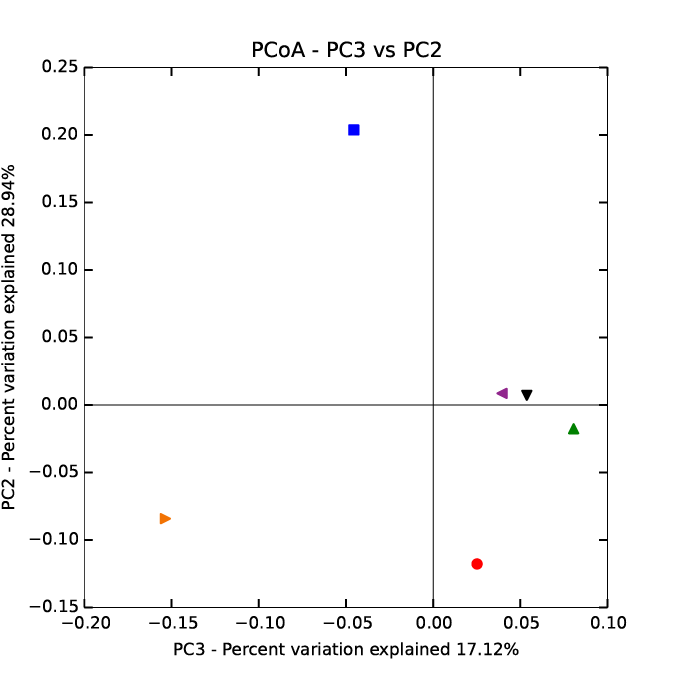

Supplement: S8 Fig — (a) PCA analysis based on PC1 vs PC2, (b) PCA analysis based on PC1 vs PC3, (c) PCA analysis based on PC3 vs PC2; 0 week (●), 1 week (▪), 2 week(▶), 3week (▲),4week (◀), 5 week (▼). (DOCX) [file pone.0233042.s008.docx]
