# Supplementary material for: Pilot plant study on nitrogen and phosphorus removal in marine wastewater by marine sediment with sequencing batch reactor
Source: PLoS One. 2020 May 19;15(5):e0233042. doi: 10.1371/journal.pone.0233042 (PMC7236998; doi:10.1371/journal.pone.0233042)
Supplement: S1 Table — (DOCX) [file pone.0233042.s009.docx]

**S1 Table.** Comparative analytical data of COD_Cr_, T-N, T-P between eco-friendly high efficiency marine sludge (eco-HEMS) and aerobic granule sludge (AGS) application in pilot plant-scale SBR treatment system during all operation period.

| Season | | | Spring & Autumn | | Summer | | Winter | |
| --- | --- | --- | --- | --- | --- | --- | --- | --- |
| Applying Sludge | | | eco-HEMS | AGS | eco-HEMS | AGS | eco-HEMS | AGS |
| COD_Cr_  (mg L^-1^) | Inf. | AVE | **149** | **154.2** | **100.8** | **163.5** | **158.0** | **164.8** |
|  |  | (min-MAX) | 109.2-210.7 | 124.5-175.6 | 63.6-150.5 | 132.2-222.2 | 85.0-311.0 | 109.2-210.7 |
|  | Eff. | AVE | **35.3** | **30.4** | **47.1** | **63.4** | **51.3** | **52.7** |
|  |  | (min-MAX) | 26.2-46.2 | 18.9-45.2 | 25.5-65.0 | 31.2-99.1 | 38.5-99.0 | 22.0-83.0 |
|  | RE (%) | AVE | **75.8** | **80.2** | **51.2** | **61.3** | **65.5** | **67.4** |
|  |  | (min-MAX) | 62.0-82.0 | 71.6-88.0 | 12.7-75.7 | 34.7-80.2 | 44.3-78.2 | 48.4-86.9 |
| T-N (mg L^-1^) | Inf. | AVE | **9.3** | **7.9** | **5.9** | **9.1** | **9.7** | **6.7** |
|  |  | (min-MAX) | 7.4-11.8 | 5.2-10.8 | 2.4-13.5 | 7.1-11.9 | 3.8-19.3 | 3.5-10.4 |
|  | Eff. | AVE | **4.2** | **3.4** | **1.5** | **6.7** | **2.0** | **3.1** |
|  |  | (min-MAX) | 2.5-6.5 | 1.8-4.5 | 0.0-4.5 | 3.2-10.4 | 0.0-6.1 | 1.6-5.1 |
|  | RE (%) | AVE | **54.9** | **55.6** | **70.9** | **26.5** | **82.0** | **53.3** |
|  |  | (min-MAX) | 38.6-74.2 | 36.8-73.1 | 4.2-100 | 3.7-65.2 | 16.6-100 | 32.3-79.8 |
| T-P  (mg L^-1^) | Inf. | AVE | **1.8** | **1.6** | **1.8** | **1.9** | **2.0** | **1.6** |
|  |  | (min-MAX) | 1.2-3.0 | 1.1-2.1 | 0.7-3.4 | 1.3-2.6 | 0.2-4.3 | 1.1-2.3 |
|  | Eff. | AVE | **0.6** | **0.5** | **1.0** | **1.2** | **0.5** | **0.8** |
|  |  | (min-MAX) | 0.4-0.9 | 0.2-1.8 | 0.0-1.9 | 0.2-1.9 | 0.0-2.9 | 0.2-1.8 |
|  | RE (%) | AVE | **65.5** | **67.9** | **47.9** | **36.7** | **79.7** | **52.5** |
|  |  | (min-MAX) | 47.1-83.3 | 0.0-88.9 | 14.3-100 | 5.3-88.2 | 27.3-100 | 9.1-90.9 |
