# Supplementary material for: Pilot plant study on nitrogen and phosphorus removal in marine wastewater by marine sediment with sequencing batch reactor
Source: PLoS One. 2020 May 19;15(5):e0233042. doi: 10.1371/journal.pone.0233042 (PMC7236998; doi:10.1371/journal.pone.0233042)
Supplement: Attachment — Submitted filename: View Letter.docx [file pone.0233042.s012.docx]

Dear Kate Armal

Thank you for your concern,

I confirm the following statements are complete and accurate. We revised the manuscript including the following statement.

Financial Disclosure:

Financial support for this project came from a grant (NRF-2015M3A9B8029697) and (NRF-2017M3A9B8065734) from National Research Foundation of Korea. Jinsoo Kim and Sungchul Kim are employed by and receive salary from Research & Development Institute of Inventory Co. Ltd.

Competing Interests:

Jinsoo Kim and Sungchul Kim are employed by and receive salary from Research & Development Institute of Inventory Co. Ltd. The specific roles of these authors are articulated in the ‘author contributions’ section. This does not alter our adherence to PLOS ONE policies on sharing data and materials

Thank you again and best regards,

Sincerely,

Sang-Seob Lee

Professor,

Department of Life Sciences

Kyonggi University

Dear Dr. Arumugam Sundaramanickam,

Thank you for giving me the opportunity to submit a revised draft of my manuscript titled “ PONE-D-19-18032: Pilot Plant Study on Nitrogen and Phosphorus Removal in Marine Wastewater by Marine Sediment with Sequencing Batch Reactor” to *PLOS ONE*. We appreciate the time and effort that you and the reviewers have dedicated to providing your valuable feedback on our manuscript. We are grateful to the reviewers for their insightful comments on our paper. We have been able to incorporate changes to reflect most of the suggestions provided by the reviewers. We have attached followings: your letter and a point-by point response to the reviewers’ comments and concerns.

Sincerely,

Sang-Seob Lee

Professor,

Department of Life Sciences

Kyonggi University

**View Letter**

| **Date:** | Nov 19 2019 09:13AM |
| --- | --- |
| **To:** | "Sang-Seob Lee" sslee@kyonggi.ac.kr |
| **cc:** | fish_lar@yahoo.com, sundarcasmb04@gmail.com |
| **From:** | "PLOS ONE" plosone@plos.org |
| **Subject:** | PLOS ONE Decision: Revision required [PONE-D-19-18032] |

PONE-D-19-18032
Pilot Plant Study on Nitrogen and Phosphorus Removal in Marine Wastewater by Marine Sediment with Sequencing Batch Reactor
PLOS ONE

Dear Professor Lee,

Thank you for submitting your manuscript to PLOS ONE. After careful consideration, we feel that it has merit but does not fully meet PLOS ONE’s publication criteria as it currently stands. Therefore, we invite you to submit a revised version of the manuscript that addresses the points raised during the review process.

We would appreciate receiving your revised manuscript by Jan 03 2020 11:59PM. When you are ready to submit your revision, log on to https://www.editorialmanager.com/pone/ and select the 'Submissions Needing Revision' folder to locate your manuscript file.

If you would like to make changes to your financial disclosure, please include your updated statement in your cover letter.

To enhance the reproducibility of your results, we recommend that if applicable you deposit your laboratory protocols in protocols.io, where a protocol can be assigned its own identifier (DOI) such that it can be cited independently in the future. For instructions see: <http://journals.plos.org/plosone/s/submission-guidelines#loc-laboratory-protocols>

Please include the following items when submitting your revised manuscript:

- A rebuttal letter that responds to each point raised by the academic editor and reviewer(s). This letter should be uploaded as separate file and labeled 'Response to Reviewers'.
- A marked-up copy of your manuscript that highlights changes made to the original version. This file should be uploaded as separate file and labeled 'Revised Manuscript with Track Changes'.
- An unmarked version of your revised paper without tracked changes. This file should be uploaded as separate file and labeled 'Manuscript'.

Please note while forming your response, if your article is accepted, you may have the opportunity to make the peer review history publicly available. The record will include editor decision letters (with reviews) and your responses to reviewer comments. If eligible, we will contact you to opt in or out.

We look forward to receiving your revised manuscript.

Kind regards,

Arumugam Sundaramanickam, PhD
Academic Editor
PLOS ONE

Journal Requirements:

When submitting your revision, we need you to address these additional requirements.

Please ensure that your manuscript meets PLOS ONE's style requirements, including those for file naming. The PLOS ONE style templates can be found at

http://www.journals.plos.org/plosone/s/file?id=wjVg/PLOSOne_formatting_sample_main_body.pdf and http://www.journals.plos.org/plosone/s/file?id=ba62/PLOSOne_formatting_sample_title_authors_affiliations.pdf

Response: Thank you for your comments information. Therefore, we revised the manuscript as PLOS ONE’s style.

**1.** Thank you for including your competing interests statement; "The authors have declared that no competing interests exist."

We note that one or more of the authors are employed by a commercial company:

"Research & Development Institute of Inventory Co. Ltd"

Response: Thank you for your comments. ‘R&D institute of Inventory Co. Ltd’ did not play a role with any funding. Also, The authors, Jinsoo Kim and Sungchul Kim, are not employed by Inventory Co. Ltd any more. Thus, we decided to erase the affiliation ‘R&D institute of Inventory Co. Ltd’ but remained the affiliation ‘Kyonggi university’. We are not related to the following comments “1, 2”.

1. Please provide an amended Funding Statement declaring this commercial affiliation, as well as a statement regarding the Role of Funders in your study. If the funding organization did not play a role in the study design, data collection and analysis, decision to publish, or preparation of the manuscript and only provided financial support in the form of authors' salaries and/or research materials, please review your statements relating to the author contributions, and ensure you have specifically and accurately indicated the role(s) that these authors had in your study. You can update author roles in the Author Contributions section of the online submission form.

Please also include the following statement within your amended Funding Statement.

“The funder provided support in the form of salaries for authors [insert relevant initials], but did not have any additional role in the study design, data collection and analysis, decision to publish, or preparation of the manuscript. The specific roles of these authors are articulated in the ‘author contributions’ section.”

If your commercial affiliation did play a role in your study, please state and explain this role within your updated Funding Statement.

  2. Please also provide an updated Competing Interests Statement declaring this commercial affiliation along with any other relevant declarations relating to employment, consultancy, patents, products in development, or marketed products, etc.

Within your Competing Interests Statement, please confirm that this commercial affiliation does not alter your adherence to all PLOS ONE policies on sharing data and materials by including the following statement: "This does not alter our adherence to PLOS ONE policies on sharing data and materials.” (as detailed online in our guide for authors <http://journals.plos.org/plosone/s/competing-interests>) . If this adherence statement is not accurate and  there are restrictions on sharing of data and/or materials, please state these. Please note that we cannot proceed with consideration of your article until this information has been declared.

Please include both an updated Funding Statement and Competing Interests Statement in your cover letter. We will change the online submission form on your behalf.

Please know it is PLOS ONE policy for corresponding authors to declare, on behalf of all authors, all potential competing interests for the purposes of transparency. PLOS defines a competing interest as anything that interferes with, or could reasonably be perceived as interfering with, the full and objective presentation, peer review, editorial decision-making, or publication of research or non-research articles submitted to one of the journals. Competing interests can be financial or non-financial, professional, or personal. Competing interests can arise in relationship to an organization or another person. Please follow this link to our website for more details on competing interests: <http://journals.plos.org/plosone/s/competing-interests>

**2.** Please include captions for your Supporting Information files at the end of your manuscript, and update any in-text citations to match accordingly. Please see our Supporting Information guidelines for more information: <http://journals.plos.org/plosone/s/supporting-information>.

Response: Thank you for your comments and we included captions for supporting information
files at the manuscript and update in-text citations to match accordingly as you commented and gave information.

**3.** PLOS requires an ORCID iD for the corresponding author in Editorial Manager on papers submitted after December 6th, 2016. Please ensure that you have an ORCID iD and that it is validated in Editorial Manager. To do this, go to ‘Update my Information’ (in the upper left-hand corner of the main menu), and click on the Fetch/Validate link next to the ORCID field. This will take you to the ORCID site and allow you to create a new iD or authenticate a pre-existing iD in Editorial Manager. Please see the following video for instructions on linking an ORCID iD to your Editorial Manager account: https://www.youtube.com/watch?v=_xcclfuvtxQ

Response: Thank you for your comments and the corresponding author ‘Sang-Seob Lee’ has updated information with ORCID ID.

Additional Editor Comments (if provided):

Dear Sang-Seob Lee,
Thank you for sending us your article and giving us the chance to consider your work. Your article was carefully reviewed by external reviewers for the consideration of publication in PLOS ONE. Unfortunately, the manuscript was not able to accept in the present form. However, we consider your article after complete revision of your manuscript. You are advised to address the comments raised by the reviewers and revise your manuscript. I strongly encourage you to seek the support of a native English speaker language expert and advised to make a new version of your manuscript.

Response: Thank you for your review. We revised the manuscript as reviewer’s comments, and we have completed the revision of the manuscript with correction and improving English. We also have taken a English editing service from Editage company, which is a recommended English editing company by PLOSONE.

[Note: HTML markup is below. Please do not edit.]

Reviewers' comments:

Reviewer's Responses to Questions

**Comments to the Author**

1. Is the manuscript technically sound, and do the data support the conclusions?

The manuscript must describe a technically sound piece of scientific research with data that supports the conclusions. Experiments must have been conducted rigorously, with appropriate controls, replication, and sample sizes. The conclusions must be drawn appropriately based on the data presented.

Reviewer #1: Yes

Reviewer #2: Yes

Reviewer #3: No

Response: Thank you for your review. We revised the conclusion from obtained data in the study.

2. Has the statistical analysis been performed appropriately and rigorously?

Reviewer #1: I Don't Know

Reviewer #2: Yes

Reviewer #3: No

Response: Thank you for your responses. We rechecked the statistical analysis in the study and revised the manuscript.

3. Have the authors made all data underlying the findings in their manuscript fully available?

The [PLOS Data policy](http://www.plosone.org/static/policies.action#sharing) requires authors to make all data underlying the findings described in their manuscript fully available without restriction, with rare exception (please refer to the Data Availability Statement in the manuscript PDF file). The data should be provided as part of the manuscript or its supporting information, or deposited to a public repository. For example, in addition to summary statistics, the data points behind means, medians and variance measures should be available. If there are restrictions on publicly sharing data—e.g. participant privacy or use of data from a third party—those must be specified.

Reviewer #1: Yes

Reviewer #2: Yes

Reviewer #3: Yes

 Response: Thank you for your review.

4. Is the manuscript presented in an intelligible fashion and written in standard English?

PLOS ONE does not copyedit accepted manuscripts, so the language in submitted articles must be clear, correct, and unambiguous. Any typographical or grammatical errors should be corrected at revision, so please note any specific errors here.

Reviewer #1: No

Reviewer #2: Yes

Reviewer #3: No

Response: Thank you for your review. We revised the manuscript as reviewer’s comments, and we have completed the revision of the manuscript with correction and improving English. We also have taken a English editing service from Editage company, which is a recommended English editing company by PLOSONE.

5. Review Comments to the Author

Please use the space provided to explain your answers to the questions above. You may also include additional comments for the author, including concerns about dual publication, research ethics, or publication ethics. (Please upload your review as an attachment if it exceeds 20,000 characters)

Reviewer #1: This article reports on the results of a pilot plant study that treats agricultural marine wastewater via a sequencing batch reactor. This seems to be the follow up to a previous laboratory scale SBR study. Overall, I find the study to be of high interest and overall it was well done. The manuscript is organized well. The main problem I see is that the authors are not specific with regards to what was done in replicate analysis. I strongly advise that the authors be clear in the methods with what kind of replicates was done.

Response: Thank you for your review. We performed analysis every day during operation of pilot-plant scale SBR system, moreover, we commented the replicates for the experiment data as well as methods in detail.

The authors should be more clear in the introduction or abstract that this builds on their previous study.

- Thank you for your comments, we make clear in the introduction or abstract about the previous study.

I am not a fan of the ‘eco’ part of the ‘eco-HEMS’ name – it seems a little bit like salesmanship and all wastewater treatment can be called ‘eco-friendly’. However, since the term was used in prior studies, the authors could also consider keeping it for consistency purposes.

- Thank you for your comments, indeed, ‘eco-HEMS’ does not imply as commercial purpose, but it emphasizes the meaning of ‘eco-friendly’ and ‘high efficiency’. We used marine sediment and high efficiency bacteria, so we wanted to use both meaning and abbreviate the words, ‘eco-friendly high efficiency marine sludge (eco-HEMS).

Though I had no problems understanding the authors, there are many areas in which grammar could be improved (I point to some cases in details below), especially in the abstract. The authors and should carefully review the final version for grammar, especially since PLOSONE won’t help. I hope the authors find my comments on some of these grammar errors are helpful.

- Thank you for your comments, we tried to fix English grammar and revised the manuscript. Indeed, we had confirmed English by Editage company, which is a recommended English editing company by PLOSONE.

I think the authors could be more specific with how they determined “optimal operational conditions”. What was the basis of ‘optimal’, and what variables were tested?

Specific comments:
Line 2. Is it more difficult because of the salinity or do we just know less about it. I think it is a reach to say it is difficult, since whole oceans are out there with all sorts of P and N cycling processes being done on a massive scale. I recommend the authors stating something more like ”Effective biological treatment of marine wastewater is not well-known.”

-> I agree with you and thank you for your comments.
We revised the sentence as you recommended.

Line 2-3. The grammar on this sentence is odd. “Accumulation of nitrogen and phosphorus from land-based effluent is a crucial cause of red-tide in marine systems” makes more sense.

-> I learned from you so much.
Thank you for your comments and we revised the sentence as you recommended.

Line 4: Add “The” before purpose

-> Thank you for your comments and we revised the sentence as you recommended.

Line 5. “a” instead of “the” before “pilot plant-scale sequencing…”

-> Thank you for your comments and we revised the sentence as you recommended.

Line 6. After the common, edit to “ elucidate which bacterial strains in sludge from marine sediment influence the performance of the SBR”.

-> Thank you for your comments and we revised the sentence as you recommended.

Line 9: “Improved by” doesn’t really make sense, maybe delete it?

-> Thank you for your comments and we delete “improved by” as you recommended.

Line 11. After summer, use a period and start a new sentence with “moreover”.

-> Thank you for your comments and we revised it as you recommended.

Line 12: “Unlike the common treatment” would be better grammar, and what is the common treatment? Please specify here so that the reader knows the main point of reference.

-> Thank you for your comments and we revised the sentence as you mentioned. Biological treatment efficiency is lower in winter because general bacteria activity is lower at the lower temperature.

Line 16: This isn’t specific enough, please state the method. (i.e. state something like “From 16S rRNA amplicon sequencing, the predominant genera was found to be ….” ). Too often, scientists are becoming careless with regards to what high throughput sequencing data gives us. If there is a PCR amplification, you don’t have a ‘true’ relative abundance, what you have a messy estimate. Thus, it needs to be clearer when we scientists are getting composition estimates based on sequencing of amplicons, vs when we are using a method that gives us a much better estimate of relative abundance (like from total rRNA direct sequencing, or true metagenomics sequencing, etc).

Line 28. Inland aquaculture and fish production may have cause HAB in some marine coastal regions, but the way this is stated grammatically implied is the main cause, which I don’t think is true.
-> Thank you for your comments and we revised the sentence.

Line 33: This needs revised, it reads that government and scientists are being monitored! A suggested edit would be “In recent decades, scientific and government monitoring in South Korea have found that increasing…”
-> Thank you for your comments and we revised the sentence as you suggested.

Line 36: A simplification would be “important in a long-term prevention strategy.”
-> Thank you for your comments and we revised the sentence as you mentioned.

Line 37. This sentence should be edited as well. “Treatment of marine wastewater needs better understood because the …..”
-> Thank you for your comments and we revised the sentence as you mentioned.

Line 60: Suggested edit “…to study possible application of …”
-> Thank you for your comments and we revised the sentence as you mentioned.

Line 61. I suggest deleting “more”
-> Thank you for your comments and we deleted it as you mentioned.

Line 71: use “a” instead of “the” to refer to the land based fish farm.
-> Thank you for your comments and we revised it as you mentioned.

Line 72: Replace “with a” instead of “and the” before pilot plant SBR. I think everything in that sentence after SBR can be deleted.
-> Thank you for your comments and we revised it as you mentioned.

Line 74. Put a period after (eco-HEMS), and start “we” as a new sentence.
-> Thank you for your comments and we revised it as you mentioned.

Line 75. “in bacterial community” is redundant information and can be deleted.
-> Thank you for your comments and we deleted it as you mentioned.

Line 76. Delete ”and predominated”
-> Thank you for your comments and we deleted the sentence as you mentioned.

Line 86 and 88. Instead of “is efficient remove”, just write “efficiently removes”
-> Thank you for your comments and we revised it as you mentioned.

Lind 107. “Was” instead of “ware” and the rest of this sentence needs some help (the MLSS was chosen based on the lab scale reactors?)
-> Thank you for your comments and we revised it as you mentioned. 1,500 mg L^-1^ of MLSS showed the highest efficiencies in the previous lab-scale reactors study.

Line 180. “as the control” doesn’t make sense to me, I would just delete these words.
-> Thank you for your comments and we deleted it as you mentioned.

Line 326. “stable” can be deleted
-> Thank you for your comments and we deleted it as you mentioned.

Line 340. This sentence is confusing.
-> Thank you for your comments and we revised the sentence as you mentioned.

Line 343. Instead of “effective bacteria”, can you use a more specific term (i.e. genera names? Are these the added bacteria?
-> Thank you for your comments and we added bacteria names (Bacillus sp. and Vibrio sp.).
 Yes, we added these bacteria for improving efficiency.

Line 349. Is this also week 1?
-> Yes, the result was also week 1.

Line 350. The plural of genus is genera.
-> Thank you for your comments and we revised it as you mentioned.

How many reads were gathered in these samples? The read depth should be stated somewhere.

Line 389-390. This sentence is confusing and needs edited.
-> Thank you for your comments and we revised it.

Line 398. Not sure how anammox connects to the information preceeding this sentence. The Anammox bacteria are Planctomycetes, which was observed (as reported in lines 379 – 9.9% Planctomycetes is actually relatively high for a non anammox reactor). Thus, I am not sure why they also say that they did not shown in the bacterial community data, unless these Planctomycetes are all from classes not known to have anammox, in which case, this should be better stated.
-> Thank you for your comments and we re-stated the results.

Reviewer #2: The baits in mariculture industry bring a lot of nutrients to the offshore area and destroy the balance of the marine ecosystem, causing red tide and other phenomena. An efficient device has been developed in this study to deal with nutrients such as N and P. This study has a high application value. The research methods and results of this paper are very detailed and logical. I think it is suitable for publication in this journal. There is only one suggestion, i.e. almost all the text in “results and discussion” is only “results”, there is almost no “discussion”. For example, what causes seasonal changes in microbial diversity? How tolerant are these strains to the different environment? I think the readers are also very interested in these mechanisms. However, mechanisms may be difficult to explain because it is not the focus of this research. Anyway, it is worthy to make more discussion.

-> Thank you so much for your comments. We added more discussion including your comments as your brilliant comment.

Reviewer #3: Summary of the manuscript
In this paper the overall idea was really good, but the methods were doesn’t have clarity and not well organized. Manuscript has lots of short comings and more related discussions should be included. It can’t be considered for the publication in the same format.
-> Thank you so much for your comments. We added more discussion including your comments as your brilliant comment.

Specific corrections
The manuscript needs an extensive review of the English language, manuscript format and also spelling mistakes.

-> Thank you for your comments and we revised and checked many mistakes and English problems.

Authors have mentioned that 89.3% & 94.9% removal during summer and 84% & 88% during winter, but stated that higher removal observed during winter. Avoid controversy statements.

Avoid earlier study’s results discussions in the methodology part (line no. 89-93); it can be included in the introduction or discussion part.

-> Thank you for your comments and we included the previous methodology and results into the introduction as you commented.

The authors stated that they used the bacterial strains of Bacillus sp. KGN1and Vibrio sp. KGP1 for the treatment of TN and TP. Since, these strains are very potential why the authors are not taken any effort to confirm the species?

-> Thank you for your comments. Indeed, we already identified the strains in the earlier previous study.

Supporting evidence for the seasonal fluctuation of environmental parameters is missing Parameters such as Temperature, Salinity and pH are closely related parameter but in the present study, authors have not observed any correlation between the parameters. The study can be improved by using statistical tools
The salinity high during winter and was decreased during spring and summer, how it possible?
Discussion part to be improved

-> Thank you for your comments. We revised and improved the environmental parameters’ part as you commented.

The references are not in the correct format; lots of punctuations errors are noticed.

 -> Thank you for your comments. We corrected the references as you commented

6. PLOS authors have the option to publish the peer review history of their article ([what does this mean?](https://journals.plos.org/plosone/s/editorial-and-peer-review-process#loc-peer-review-history)). If published, this will include your full peer review and any attached files.


If you choose “no”, your identity will remain anonymous but your review may still be made public.


**Do you want your identity to be public for this peer review?** For information about this choice, including consent withdrawal, please see our [Privacy Policy](https://www.plos.org/privacy-policy).

Reviewer #1: No

Reviewer #2: No

Reviewer #3: No


[NOTE: If reviewer comments were submitted as an attachment file, they will be attached to this email and accessible via the submission site. Please log into your account, locate the manuscript record, and check for the action link "View Attachments". If this link does not appear, there are no attachment files to be viewed.]


While revising your submission, please upload your figure files to the Preflight Analysis and Conversion Engine (PACE) digital diagnostic tool, <https://pacev2.apexcovantage.com/>. PACE helps ensure that figures meet PLOS requirements. To use PACE, you must first register as a user. Registration is free. Then, login and navigate to the UPLOAD tab, where you will find detailed instructions on how to use the tool. If you encounter any issues or have any questions when using PACE, please email us at [figures@plos.org](mailto:figures@plos.org). Please note that Supporting Information files do not need this step.

*In compliance with data protection regulations, you may request that we remove your personal registration details at any time.* [*(Remove my information/details)*](https://www.editorialmanager.com/pone/login.asp?a=r)*. Please contact the publication office if you have any questions.*
